# Application of Microbial Fermentation in Caffeine Degradation and Flavor Modulation of Coffee Beans

**DOI:** 10.3390/foods14152606

**Published:** 2025-07-24

**Authors:** Lu-Xia Ran, Xiang-Ying Wei, Er-Fang Ren, Jian-Feng Qin, Usman Rasheed, Gan-Lin Chen

**Affiliations:** 1 Guangxi Subtropical Crops Research Institute, Guangxi Academy of Agricultural Sciences, Nanning 530001, China; 1211016008@gnnu.edu.cn (L.-X.R.); 15102932953@163.com (X.-Y.W.); aabbc159@163.com (E.-F.R.); qjfmail@126.com (J.-F.Q.); rasheus@outlook.com (U.R.); 2 Key Laboratory of Quality and Safety Control for Subtropical Fruit and Vegetable, Ministry of Agriculture and Rural Affairs, Nanning 530001, China; 3 Guangxi Key Laboratory of Quality and Safety Control for Subtropical Fruits, Nanning 530001, China

**Keywords:** decaffeination, low-caffeine coffee, microbial fermentation, mixed-culture, flavor enhancement

## Abstract

Coffee is one of the most widely consumed beverages worldwide, primarily due to the stimulating effects attributed to its caffeine content. However, excessive intake of caffeine results in negative effects, including palpitations, anxiety, and insomnia. Therefore, low-caffeine coffee has captivated growing consumer interest, highlighting its significant market potential. Traditional decaffeination methods often lead to non-selective extraction, resulting in a loss of desirable flavor compounds, thereby compromising coffee quality. In recent years, microbial fermentation has emerged as a promising, targeted, and safe approach for reducing caffeine content during processing. Additionally, mixed-culture fermentation further enhances coffee flavor and overcomes the drawbacks of monoculture fermentation, such as low efficiency and limited flavor profiles. Nonetheless, several challenges are yet to be resolved, including microbial tolerance to caffeine and related alkaloids, the safety of fermentation products, and elucidation of the underlying mechanisms behind microbial synergy in co-cultures. This review outlines the variety of microorganisms with the potential to degrade caffeine and the biochemical processes involved in this process. It explores how microbes tolerate caffeine, the safety of metabolites produced during fermentation, and the synergistic effects of mixed microbial cultures on the modulation of coffee flavor compounds, including esters and carbonyls. Future directions are discussed, including the screening of alkaloid-tolerant strains, constructing microbial consortia for simultaneous caffeine degradation for flavor enhancement, and developing high-quality low-caffeine coffee.

## 1. Introduction

Coffee is one of the most widely consumed beverages in the world, prized for its rich flavor and invigorating effects. These attributes stem from the unique chemical composition of coffee beans, which includes chlorogenic acids, lipids, proteins, amino acids, and alkaloids like caffeine and trigonelline [1]. Among these, alkaloids are closely linked to the quality of coffee. Caffeine, the primary methylxanthine alkaloid in coffee, exerts psychoactive effects that enhance alertness, elevate mood, and sharpen mental focus [2], suggesting its key role in consumers’ preference for coffee selection. Caffeine has also been shown to promote energy expenditure and fat oxidation [3], as well as improve vigilance and visual attention. However, caffeine exhibits the aforementioned effects in a dose-dependent manner. High caffeine intake (≥400 mg/day) is associated with anxiety [4,5], insomnia, irritability, restlessness [6], and tachycardia or arrhythmias, particularly in caffeine-sensitive individuals. Additionally, caffeine withdrawal in habitual users can lead to headaches, fatigue, reduced alertness, and depressed mood [7]. As consumer health awareness increases and coffee demand rises, there is a substantial demand for low-caffeine coffee. Therefore, addressing the challenge of caffeine reduction without compromising product quality stands as the primary objective for the coffee processing industry.

Current decaffeinated coffee products available in the market include industrially processed, naturally low-caffeine, and genetically modified varieties [8]. Industrial decaffeinated coffee is the most common type and is typically produced using methods like the Swiss Water Process and solvent extraction. However, available techniques may alter the flavor profile or leave chemical residues in the final product. Supercritical carbon dioxide extraction is a safer and more efficient method, although it involves high operational costs. Naturally low-caffeine coffee varieties are typically not suited for large-scale commercialization due to their low yields and inadequate disease resistance. Genetically modified low-caffeine coffee has potential but faces consumer acceptance issues due to health concerns. As a result, developing novel decaffeination strategies has become a research priority [9]. Controlled microbial fermentation has emerged as a promising technology. It offers both caffeine reduction and flavor enhancement by degrading mucilage layers and producing bioactive compounds while also inhibiting pathogenic microorganisms. Microorganisms capable of caffeine degradation have primarily been isolated from soils in coffee and tea plantations, including *Aspergillus sydowii* [10], *Pseudomonas putida* [11], and *Pseudomonas alcaligenes* CFR 1708 [12]. Currently, these microorganisms are mainly employed in tea fermentation [10,13,14], but not in coffee bean fermentation. Microbial fermentation not only reduces the stimulating effects of caffeine but also enhances the sensory quality of coffee through metabolite optimization [15]. Incorporating microbes that degrade caffeine into coffee fermentation offers a feasible and sustainable method for producing safer decaffeinated coffee products.

Fermentation is a common method used in coffee production and is essential for developing flavor. Natural fermentation has proven successful in fruit wine production, giving fermented products their unique flavors [16]. Microbial fermentation produces low-caffeine coffee, combining ‘low caffeine’ with natural processing. The combination of low irritation and natural flavor appeals to health-conscious consumers. As coffee demand rises, applying this fermentation process to the coffee industry is expected to gain widespread consumer approval. During fermentation, sugars and proteins are broken down, producing acids, esters, and other compounds. These compounds contribute to the richness and flavor complexity of the coffee. Monoculture fermentation provides better process control and results in consistent product quality, but carries significant limitations. These include simplified metabolic pathways, slower fermentation rates, reduced ester production, and a relatively bland flavor profile [17]. Additionally, external carbon sources like glucose or sucrose are often needed to promote the growth of individual strains, which raises production costs. Because of these constraints, co-culture fermentation is gaining popularity in coffee processing. Interactions among different microbial species can enhance fermentation efficiency, reduce processing time, and help maintain or improve the quality of coffee. These microbes interact with flavor precursor compounds in coffee beans to produce esters, ketones, alcohols, acids, and aldehydes. When roasted, these compounds contribute to the coffee’s distinctive aroma [18]. Mixed fermentation allows for greater substrate utilization, reduces by-product formation, and enhances resource efficiency. Moreover, co-cultures exhibit broader environmental adaptability and can produce a wider range of volatile compounds associated with fruity and caramel-like aromas [19].

The co-inoculation of lactic acid bacteria (LAB) and yeasts is a widely used strategy in coffee fermentation that significantly enhances acidity, fruitiness, and overall sensory quality [1,20]. To prevent interspecies competition for nutrients and reduce possible antagonistic interactions, sequential inoculation is commonly used in mixed fermentations. This technique is widely used in the production of fruit wine [21] and dairy fermentations [22], allowing early inoculated microbes to condition the environment for subsequent strains. The timing of inoculation is crucial for the sensory quality of the fermented product. In addition, proper control of environmental conditions during fermentation is essential for achieving desirable coffee flavors while minimizing unwanted off-notes.

This work evaluates the merits of different low-caffeine coffee production methods, emphasizing the diversity and metabolic processes of microbes that degrade caffeine. It further examines how mixed-strain fermentation can improve flavor and aroma and discusses future research directions and applications aimed at enhancing the appeal of fermented low-caffeine coffee.

## 2. Caffeine and Decaffeinated Coffee

Coffee contains a range of bioactive compounds, including alkaloids like caffeine, theobromine, trigonelline, and theophylline. It also contains polyphenols, such as chlorogenic acids, flavonoids, coumarins, and quercetin, along with carbohydrates, amino acids, proteins, and lipids. These components play a crucial role in developing flavor and are significantly influenced by factors such as coffee variety, geographic origin, and harvest time.

Caffeine is the most widely consumed psychoactive substance worldwide, present in the daily diets of about 80% of the global population through various products [23]. In Asia, tea is the main source of caffeine, while in Western countries such as those in Europe and North America, coffee is the primary source. As a natural central nervous system stimulant, caffeine exerts a notable alertness-enhancing effect. Its primary mechanism involves mimicking the structure of adenosine, which competitively inhibits adenosine from binding to its receptors—a process that typically promotes drowsiness. Caffeine also increases the secretion of dopamine, which boosts alertness and improves response time [2,3].

In addition to its stimulant effects, coffee has demonstrated antioxidant, anti-inflammatory, neuroprotective, and anticancer properties, which help support cognitive function and memory preservation [24]. It is important to note that caffeine does not eliminate fatigue; it temporarily masks it by stimulating energy expenditure. However, excessive or chronic use may lead to health concerns. While many studies support caffeine’s role in enhancing alertness, prolonged high-dose consumption can lead to dependence and negative effects, including palpitations, headaches, sleep disturbances, anxiety, gastrointestinal discomfort, and arrhythmias [5]. The definition of “excessive intake” is influenced by individual factors such as age, sex, and overall health status, and regular consumers may develop a tolerance to caffeine’s stimulatory effects. Recent studies indicate that chronic caffeine consumption may hinder vascular dilation and irritate the gastrointestinal tract. In contrast, decaffeinated coffee is regarded as more suitable for individuals with cardiovascular or digestive disorders [25]. As awareness of caffeine’s health effects increases, the demand for decaffeinated coffee has become more significant in the market. The European Union defines beverages with over 150 mg/L of caffeine as “high-caffeine” and sets the daily caffeine intake limit for healthy adults to no more than 400 mg [26]. In Brazil, the regulatory limit for decaffeinated roasted coffee is ≤0.1% caffeine, as mandated by the Brazilian Ministry of Agriculture, Livestock and Supply (MAPA) Ordinance No. 570 (2022).

Although decaffeinated coffee may be less appealing to avid caffeine consumers, it offers a viable alternative for caffeine-sensitive individuals and those with cardiovascular or hypertensive conditions. This broadens the scope of coffee consumption for its sensory qualities without compromising health, thereby expanding its commercial potential. Market data indicate that the United States and Germany are the largest consumers of decaffeinated coffee [27]. In 2009, the International Coffee Organization reported that over 10% of the global coffee market was made up of decaffeinated products, with the U.S. accounting for more than 16% of total coffee consumption in this category. Consumer acceptance of decaf coffee is significantly increasing; however, factors like cost, awareness, and supply chain issues still hinder its market growth. A consumer survey involving 12,098 participants revealed that 13% exclusively consumed decaffeinated coffee, while 27% consumed both caffeinated and decaffeinated varieties [28]. Initially, the decaffeinated coffee market is primarily dominated by older consumers in Western countries. As public awareness of health and wellness increases, this market is becoming more popular among younger demographics. Collectively, these findings highlight the substantial market potential and evolving consumer demand for decaffeinated coffee.

## 3. Decaffeinated Coffee Production Methods: Advantages and Limitations

### 3.1. Industrial Decaffeinated Coffee: Physical and Chemical Methods

To meet the growing demand for decaffeinated coffee, researchers have explored numerous methods for caffeine removal. Early efforts to decaffeinate coffee were ineffective until the use of steam on green coffee beans was discovered. This process increased their moisture content and internal surface area, enhancing the efficiency of caffeine extraction with organic solvents [29]. Initially, various decaffeination techniques used organic solvents such as dichloromethane, benzene, chloroform, ether, trichloroethylene, carbon tetrachloride, acetone, ammonium hydroxide, and sulfuric acid. However, many of these solvents are considered potentially hazardous compounds. The U.S. Food and Drug Administration (FDA) has set allowable limits for residual solvents in decaffeinated coffee. For instance, the level of dichloromethane must be below 10 parts per million (ppm) [30]. However, even after roasting, residual levels of 2 to 3 ppm have still been detected, which raises public health concerns. Since 1982, the FDA has approved ethyl acetate as a primary solvent for removing caffeine. Naturally found in many fruits and known for its pleasant aroma, ethyl acetate is generally recognized as safe. As a result, regulatory agencies, including the FDA, do not impose strict limits on its residue in decaffeinated coffee. Moreover, residual ethyl acetate can be effectively eliminated through steaming during post-treatment. However, it remains unclear whether ethyl acetate extraction removes other chemical constituents from the beans and alters the coffee’s flavor profile, as no conclusive studies have been reported to date.

The Swiss Water Process is a popular method for producing decaffeinated coffee. It removes caffeine from green coffee beans through selective diffusion using pure water. This method has demonstrated an efficacy of up to 99% caffeine removal while preserving the original flavor profile of the beans [31]. Beans are initially soaked in a caffeine-free green coffee extract that is rich in other compounds to facilitate the diffusion-driven decaffeination process. This method has since been improved by utilizing custom-designed activated carbon beds, which are preloaded with coffee extract or sucrose solutions. These beds selectively adsorb alkaloids, minimizing the loss of other desirable compounds in the beans.

Another commonly employed method for decaffeinating tea is hot water extraction. Liang et al. demonstrated that up to 83% of caffeine can be removed from fresh green tea using hot water, indicating the effectiveness of this method [32]. It is noteworthy that water is a non-selective solvent that often co-extracts other compounds along with caffeine, which can potentially affect the sensory quality of the final product. Additionally, the hot water extraction process produces large amounts of industrial wastewater, which raises environmental concerns.

Another safe and efficient method for removing caffeine is supercritical carbon dioxide (CO_2_) extraction [31]. In this process, pre-moistened coffee beans (typically with around 50% moisture content) are placed into a sealed stainless steel extraction vessel. Liquid CO_2_ is introduced into the system, which is then pressurized and heated to approximately 300 atm and 150 °F to achieve supercritical conditions, significantly enhancing the solubility of caffeine. An activated carbon adsorption unit is integrated into the system. As supercritical CO_2_ circulates through the beans, caffeine is selectively extracted. It is either retained on the activated carbon or transferred to a washing unit, where it is removed with water. Excess water and residual CO_2_ in the beans are subsequently eliminated through drying. This method can effectively remove over 96% of caffeine while maintaining the sensory qualities of the coffee. However, it requires specialized equipment that can handle high pressure (up to 15 MPa) and elevated temperatures (around 70 °C). Additionally, it utilizes large volumes of CO_2_ and a complex recovery system, thereby elevating the operational costs. Several patented improvements have been proposed to address these limitations, including (i) replacing CO_2_ with nitrous oxide (N_2_O), which has higher caffeine solubility; (ii) acidifying the beans during the pre-wetting step to enhance coffee acidity lost during extraction; and (iii) using liquid CO_2_ at a reduced pressure instead of supercritical CO_2_ to better preservs the flavor quality of decaffeinated coffee.

In addition to conventional methods, adsorption and metal precipitation have also been employed for caffeine removal [33,34]. Adsorptive removal is primarily used for the sequestration of alkaloids from environmental wastewater. Common adsorbents include activated carbon, palm inner husk-derived activated carbon, and composite materials supported by bovine bone-based activated carbon. This method is easy to use and demonstrates high removal efficiency. Despite their benefits, adsorption materials face significant challenges, including selectivity issues, high costs, and limited lifespans. Metal precipitation takes advantage of the chemical stability of caffeine, which typically does not form strong complexes with metal ions [33]. However, this method has drawbacks, including low recovery efficiency, a substantial loss of active components, and the potential presence of chemical reagent residues.

### 3.2. Naturally Low-Caffeine and Genetically Modified Coffee

Producing decaffeinated coffee should ensure that the beverage retains its desired flavor characteristics. Consequently, research has expanded beyond just the technical removal of caffeine. It now includes traditional studies of genetic variation as well as the development of genetically modified (GM) coffee plants that are caffeine-free [35]. These naturally decaffeinated coffee species are largely unexploited due to the presence of bitter diterpene glycosides of the furokaurane type, which create undesirable flavor attributes [36]. Several genes control caffeine content in coffee beans, and it can potentially be reduced through both intra- and interspecific hybridization. Alkaloid profiling of different coffee cultivars shows that, except for the mutant cultivar Laurina, most varieties have caffeine levels between 1% and 1.3% [37]. In contrast, low-caffeine germplasm has been identified among the offspring of interspecific hybrids, where the caffeine content usually reverts to approximately 1% after several breeding generations. ‘Laurina’, a cultivar of *Coffea arabica*, is recognized for its excellent cup quality and naturally low caffeine content. However, it also has low yield potential, making it unsuitable for commercial cultivation [38]. Consequently, researchers have turned their focus toward identifying naturally occurring low-caffeine variants.

In a study of over 700 Ethiopian coffee varieties, three genotypes were identified as having remarkably low caffeine levels (0.07%) [36]. Unfortunately, the identified genotypes were not productive in agricultural terms. The only viable strategy was to transfer the low-caffeine trait into high-yielding commercial cultivars of *C. arabica* through hybridization. However, genetic incompatibility between species hindered the successful incorporation of the “absence of caffeine” trait into cultivated varieties such as *C. arabica* and *C. canephora*.

Research on the enzymatic degradation of caffeine is limited, while most studies have concentrated solely on enzymes involved in caffeine biosynthesis. The post-transcriptional suppression of key caffeine biosynthetic enzymes has significantly decreased caffeine content [39,40]. Since theobromine is crucial in caffeine production, inhibiting its synthesis effectively reduces caffeine levels. These findings lay the groundwork for developing genetically modified (GM) caffeine-free coffee.

Transgenic methods theoretically offer a way to create decaffeinated coffee. However, caffeine serves vital ecological functions in the coffee plant, as it protects young leaves and fruits from pests and pathogens while also inhibiting the growth of competing vegetation [41]. These protective functions pose significant challenges to the commercial viability and agronomic stability of caffeine-free transgenic coffee lines.

## 4. Microbial Fermentation for Caffeine Degradation

As mentioned earlier, there are several methods for removing caffeine from coffee beans. Currently, the majority of decaffeinated coffee available on the market is produced using the Swiss Water Process. However, this water-based method lacks selectivity and often extracts other compounds along with caffeine, which can negatively impact the flavor of the coffee. The supercritical carbon dioxide method is both efficient and safe; however, it requires costly equipment, which can be unaffordable for small-scale producers. On the other hand, solvent extraction is effective in removing caffeine, but it carries the risk of chemical residue. In contrast, caffeine-degrading microorganisms such as *Saccharomyces cerevisiae* and *Rhizopus oryzae* (Table 1) are easily accessible and offer an alternative solution. These microbes are frequently utilized in food fermentation to enhance flavor and have the ability to degrade caffeine. They provide a low-cost option for cultivation and can improve the flavor profile of decaffeinated coffee. As a result, using microbial fermentation for caffeine degradation offers a safer and more cost-effective method for producing decaffeinated coffee for the market.

### 4.1. Diversity and Mechanisms of Caffeine-Degrading Microorganisms

**Figure 1:**
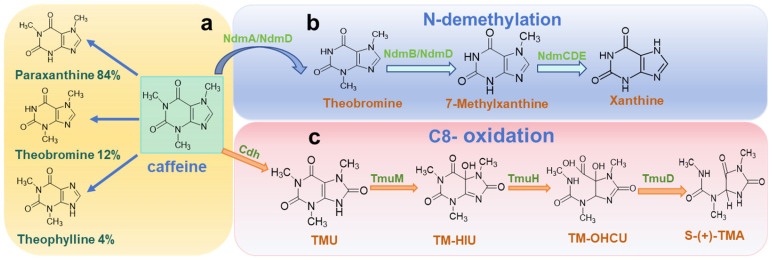
Caffeine metabolism and degradation pathways. (**a**) Caffeine metabolites. (**b**) Proposed caffeine N-demethylation pathway in *Pseudomonas putida* CBB5 [47]. (**c**) Proposed caffeine C-8 oxidation pathway in *Pseudomonas* sp. CBB1 [48]. NdmA =  N1-demethylase specific for N1-methyl group of caffeine; NdmD =  reductase; NdmB =  N3-demethylase specific for N3-methyl group of theobromine; NdmCDE  =  protein complex containing N7-demethylase specific for N7-demethylation of 7-methylxanthine; Cdh =  trimeric caffeine dehydrogenase; TmuM =  trimethyluric acid monooxygenase; TumH  =  putative TM-HIU hydrolase; TmuD  =  putative TM-OHCU decarboxylase. This figure is adapted from the references [49].

Caffeine is the main methylxanthine alkaloid found in coffee beans, which has three methyl groups located at the N-1, N-3, and N-7 positions. Stepwise demethylation of caffeine leads to the formation of theobromine, paraxanthine, and theophylline, with paraxanthine being the predominant metabolic product (Figure 1a) [14]. Conventional methods for caffeine removal include hot water extraction, adsorption separation, solvent extraction, and supercritical CO_2_ extraction. These techniques often face limitations, including solvent residue, a loss of flavor compounds, and high operational costs. In contrast, microbial fermentation provides a greener, more cost-effective alternative. Introducing caffeine-specific microbial strains can reduce caffeine content while generating flavor precursor compounds that stabilize and enhance coffee’s sensory profile, making this approach highly promising for practical applications. A review of the relevant literature and academic databases, combined with statistical data reported by Mock [42] reveals that as of April 2025, a total of 121 microorganisms have been identified with caffeine-degrading capabilities. These include 80 bacterial species, 33 fungi, and 8 yeast strains. Notably, several new caffeine-degrading strains have been identified since 2022, including *Bacillus* sp. KS38 [43], *Rhodococcus qingshengii* [44], *Trametes versicolor* [45], and *Torulaspora delbrueckii* [46].

Bacteria represent the predominant group of caffeine-degrading microorganisms, with the genus *Pseudomonas* being the most extensively studied [50,51,52]. This could be attributed to their resilience in extreme environments and their ability to metabolize alkaloids and other complex compounds. Caffeine degradation in bacteria is accomplished via two main pathways: N-demethylation and C-8 oxidation [49]. Among 37 identified strains of *Pseudomonas*, 16 have been confirmed to degrade caffeine via the N-demethylation pathway, indicating the dominance of this mechanism. In this pathway, methyl groups are sequentially removed from the nitrogen atoms of the xanthine ring, ultimately yielding xanthine as the end product (Figure 1b) [53]. In contrast, the C-8 oxidation pathway involves the activity of caffeine dehydrogenase or oxidase, which catalyzes the formation of 1,3,7-trimethyluric acid (TMU). This compound is then further degraded through a series of enzymatic reactions to produce oxaloacetate, dimethylurea, and monomethylurea (Figure 1c) [54]. Among the 37 *Pseudomonas* strains, only *Pseudomonas* sp. CBB1 has been shown to exclusively utilize the C-8 oxidation pathway [48], whereas *Pseudomonas putida* CT25 possesses both N-demethylation and C-8 oxidation capabilities [55]. Beyond the 37 *Pseudomonas* strains, 43 bacterial species have been reported to possess caffeine-degrading potential [42]. Notable examples of employing the N-demethylation pathway include *Bacillus amyloliquefaciens* HZ-12 [39], *Bacillus licheniformis* [56], *Paenibacillus macerans* [57], *Paraburkholderia caffeinilytica* CF1 [58], *Salinivibrio costicola* GL6 [59], and *Serratia marcescens* [60]. Only *Alcaligenes* sp. CF8 [61] and members of *Klebsiella* and *Rhodococcus* [62] have been shown to degrade caffeine via the C-8 oxidation route. Among the 80 known caffeine-degrading bacterial species, they can be classified into 32 genera, with 21 of these belonging to Gram-negative bacteria. This highlights a significant taxonomic bias toward Gram-negative lineages in microbial caffeine metabolism.

Research on fungal caffeine degradation is limited compared to that on bacteria. Among fungi, yeasts are the most frequently reported organisms capable of degrading caffeine. According to available data, eight yeast species have demonstrated the ability to metabolize caffeine. These include Candida inconspicua and Candida tropicalis [63], *Meyerozyma* (Pichia) *guillermondii*, *Saccharomyces boulardiilyo*, *S. cerevisiae* BY4741, and *S. cerevisiae* TFS9 [64,65], *Trichosporon asahii* [66], and *Torulaspora delbrueckii* [46]. Among these, only *Trichosporon asahii* has a clearly elucidated caffeine degradation pathway, which proceeds via N-demethylation. The metabolic mechanisms in other yeast species are largely uncharacterized and require further investigation.

In addition to yeasts, 33 types of filamentous fungi have been identified as capable of degrading caffeine. Among these, the *Aspergilli* are the most frequently reported, comprising 11 of the 33 strains. They represent the most common caffeine-degrading fungi outside of the yeast group. Other notable caffeine-degrading fungi include *Aureobasidium* sp. [67], *Chrysosporium keratinophilum* [68], *Coniophora puteana* [69], *Fusarium solani* and *Gliocladium roseum* [70], *Penicillium* sp. [13], *Phanerochaete chrysosporium* BK [71], *Pleurotus ostreatus* [72], *Stemphylium* sp. [73], *Trichophyton mentagrophytes* and *Epidermophyton floccosum* [74], *Rhizopus* sp. [75], and the basidiomycete *Trametes versicolor* [45]. These fungi have shown significant potential to degrade caffeine, with peak activity generally observed at 25 °C and under neutral pH conditions.

Among the 33 filamentous fungi, 14 species have been confirmed to degrade caffeine via the N-demethylation pathway. To date, no fungal species has been reported to utilize the C-8 oxidation pathway for caffeine degradation.

Out of the 121 identified microorganisms that degrade caffeine, more than 84% are capable of using caffeine as their primary carbon source. However, all strains require supplementation with additional sugars such as fructose, glucose, or sucrose to support optimal growth and metabolic activity. So far, 21 of these strains have been used in the fermentation of coffee, tea, or their by-products (Table 1). These include 18 different fungal strains, which consist of 7 species of *Aspergillus*, 4 species of *Rhizopus*, 4 types of yeast, 2 white-rot fungi, and 1 species of *Penicillium*, along with 3 bacterial strains. A detailed discussion regarding the safety of these microorganisms for use in food fermentation will be provided in the subsequent section.

While many bacterial and fungal strains capable of degrading caffeine have been identified, research on optimizing degradation conditions, along with the specific enzymes and genes involved in caffeine metabolism, remained unexplored. Microorganisms are known to degrade caffeine through the combined action of demethylases and oxidases. However, there are currently no studies demonstrating the use of isolated enzymes for caffeine degradation. This is likely due to the inherent instability of these enzymes under industrial conditions. Additionally, the safety of using caffeine-degrading microorganisms in food fermentation has not been thoroughly assessed. Therefore, a comprehensive investigation of the enzymes and genes associated with microbial caffeine degradation, along with rigorous safety assessments, is crucial to emphasize their practical application. These studies are essential for clarifying the molecular processes involved in microbial caffeine metabolism, which will aid in creating safe and effective low-caffeine products like decaffeinated coffee and tea.

**Table 1:** Application of caffeine degradation microorganisms in coffee, tea, and their by-products.

	Microorganism	Carbon Source	Degradation Pathway	Source/Application	Degradation Efficacy	Flavor Change	References
1	*R. oryzae* FNCC6010	—	—	Coffee Beans	0.05 mg·g^−1^·h^−1^	—	[15]
2	*S. cerevisiae*	—	—	Coffee Beans	0.018 mg·g^−1^·h^−1^	—	[15]
3	*Leuconostoc mesenteroides*	—	—	Coffee Beans	0.020 mg·g^−1^·h^−1^		[15]
4	*Lactobacillus casei*	—	—	Coffee Beans	0.020 mg·g^−1^·h^−1^		[15]
5	*Torulaspora delbrueckii* CCMA 0648	—	—	Coffee Beans	0.048 mg·g^−1^·h^−1^	Chocolate, Caramel, Honey	[46]
6	*Aspergillus ochraceus*	—	—	Coffee Beans	—	—	[76]
7	*Aspergillus* sp.	—	N-demethylation	Coffee husk	—	—	[71]
8	*Aspergillus* sp. V12A25	Caffeine + sucrose	—	Coffee grounds	6.54 mg·L^−1^·h^−1^	—	[77]
9	*Aspergillus fumigatus* C11B25	Caffeine + sucrose	—	Coffee grounds	5.00 mg·L^−1^·h^−1^	—	[77]
10	*Aspergillus niger* C16A25	Caffeine + sucrose	—	Coffee grounds	5.07 mg·L^−1^·h^−1^	—	[77]
11	*Rhizopus* sp. LPB-79	—	—	Coffee husk	—	—	[71]
12	*R. delemar*	Caffeine + sugars	N-demethylation	Coffee husk	—	—	[78]
13	*Rhizopus oryzae MUCL 28168*	_	N-demethylation	Coffee pulp	—	—	[75]
14	*Phanerochaete chrysosporium* BK	—	—	Coffee husk	—	—	[71]
15	*Pleurotus ostreatus*	—	N-demethylation	Coffee grounds	—	—	[72]
16	*Brevibacterium* sp. MTCC 10313	Glucose + Caffeine	—	Coffee Pulp	—	—	[79]
17	*Penicillium simplicissimum* 4–17	—	—	Tea	0.043 mg·g^−1^·h^−1^	like the smell of fermented soyabean	[13]
18	*Aspergillus niger* NCBT110A	glucose	—	Pu’er Tea	—	—	[14]
19	*Aspergillus sydowii* NRRL250	Caffaine	N-demethylation	Pu’er Tea	2.57 mg·L^−1^·h^−1^	—	[14]
20	*Candida famata* ACCC 2052	—	—	Black/Green Tea	—	—	[80]
21	*Candida albicans* ACCC 2100	—	—	Black/Green Tea	—	—	[80]

### 4.2. Enzymes Involved in Caffeine Degradation

Caffeine can be degraded enzymatically by specific enzymes. In *Pseudomonas putida*, five N-demethylase genes have been identified: *ndmA*, *ndmB*, *ndmC*, *ndmD*, and *ndmE*. These genes produce a group of soluble Rieske non-heme iron monooxygenases that facilitate N-demethylation reactions [47]. NdmA, NdmB, and NdmC, respectively, show demethylation at the N1, N3, and N7 sites. NdmD is crucial for the catalytic activity of all N-demethylation processes [81]. NdmE is thought to play a structural role and is essential for the soluble expression of the NdmCDE enzyme complex [82]. For specific roles, please refer to Table S1. In addition to these enzymes, other caffeine metabolism-related enzymes have been identified in the N-demethylation pathway of *P. putida*. For instance, isoxanthine demethylase catalyzes the oxidative demethylation of isoxanthine to xanthine. However, this enzyme exhibits no activity towards caffeine, indicating that each step of caffeine demethylation requires highly specific enzymes for different substrates. Furthermore, the purified isoxanthine demethylase was found to be structurally unstable [83], posing challenges for its potential application.

In the C-8 oxidation pathway of bacterial caffeine degradation (Figure 1c), caffeine is first oxidized by a dehydrogenase or oxidase to form 1,3,7-trimethyluric acid (TMU). Subsequently, a series of enzymatic reactions involving TmuM, TmuH, and TmuD convert TMU into oxaloacetate, dimethylurea, and monomethylurea [54]. For specific roles, please refer to Table S1. The C-8 oxidation pathway has also been observed in *Klebsiella* and *Rhodococcus* species, where caffeine oxidase was identified as a key enzyme [62]. A caffeine oxidase derived from *Alcaligenes* sp. was a serine-type metalloprotease specifically designed to act on caffeine, using it predominantly as its substrate [61]. While xanthine oxidase from *Pseudomonas putida* L. can oxidize xanthine, hypoxanthine, 3-methylxanthine, and xanthinol into their respective methyluric acids, it cannot metabolize caffeine [84]. A novel caffeine oxidase has been identified that specifically catalyzes the oxidation of caffeine at the C-8 position. It is also capable of converting 1-, 3-, and 7-substituted methylxanthines into their corresponding 8-oxo derivatives [85]. However, to date, no xanthine oxidase or dehydrogenase has been shown to directly metabolize caffeine, underscoring the substrate specificity and complexity of caffeine degradation pathways.

### 4.3. Metabolic Shifts in Caffeine-Degrading Fermentation

During caffeine-degrading fermentation, caffeine is demethylated to xanthine, accompanied by changes in various nutrients and volatile metabolites [86]. The growth of fermentative microbes and the production of metabolites are closely linked to whether cellular metabolism involves aerobic or anaerobic respiration. Aerobic respiration generates more ATP, CO_2_, and water, whereas anaerobic respiration produces higher levels of organic acids and volatile compounds. During the coffee fermentation process, various biochemical pathways, including alcoholic fermentation, lactic and hetero-lactic fermentation, fatty acid degradation, acetylation, and enzymatic hydrolysis, contribute to the formation of aromatic compounds and flavor precursors. These processes enhance the sensory profile of coffee beans.

**Figure 2:**
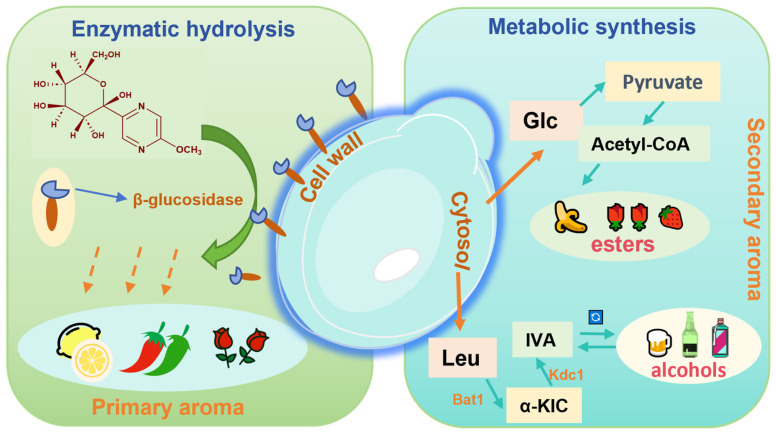
Types and pathways of yeast metabolism.

During coffee fermentation, various microbial species create unique metabolic profiles. *S. cerevisiae* is known for its ability to produce esters during caffeine degradation [79]. Yeast-driven aroma formation during fermentation occurs through two primary pathways (Figure 2): (i) the enzymatic hydrolysis of glycosidic precursors, which releases primary aroma compounds such as methoxypyrazines and terpenes, and (ii) de novo synthesis of secondary aroma compounds such as volatile fatty acids, higher alcohols, esters, and aldehydes [87]. Acetate esters are important metabolites formed by the condensation of acetyl-CoA or acetic acid with higher alcohols [88]. Volatile fatty acids such as acetic, butyric, and valeric acids are mainly produced through glycolysis, the tricarboxylic acid (TCA) cycle, and the pentose phosphate pathway [89]. In a study on the fermentation of Pu-erh tea by the caffeine-degrading fungus *Aspergillus sydowii*, the primary metabolites identified were theobromine and 3-methylxanthine. This process notably affected amino acid and flavonoid metabolism, leading to the increased accumulation of bioactive compounds such as ketoprofen, baclofen, and tolbutamide [10]. Similarly, fermentation of green tea with *Aspergillus niger* led to significant increases in alcohols, alkanes, nitroxides, soluble sugars, total flavonoids, free amino acids, and volatile compounds such as linalool, (E)-linalool oxide, linalool oxide, and theapyrrole [90].

Caffeine significantly contributes to the bitterness of coffee, enhancing its flavor complexity and mouthfeel. During coffee fermentation, the challenge lies in reducing caffeine content while preserving sensory quality and minimizing the loss of key nutrients and volatile compounds. As facultative anaerobes, yeasts are primary ester producers and possess caffeine-degrading capacity, making them suitable for co-inoculation with other caffeine-degrading microbes to improve the quality of low-caffeine coffee.

### 4.4. Screening of Caffeine-Degrading Strains

Although the caffeine-metabolizing capabilities of microbes in coffee grounds, coffee beans and tea leaves, have been explored, their tolerance to high concentrations of caffeine remains unclear. Furthermore, optimizing fermentation conditions to improve caffeine degradation efficiency is essential. Therefore, the selection of caffeine-degrading microorganisms should focus on the following aspects.

#### 4.4.1. Caffeine Degradation Capacity of the Strains

The caffeine degradation capacity of microorganisms is a key factor in the production of low-caffeine coffee. Strains with high degradation efficiency can accelerate fermentation and better preserve product quality. Unlike many other microbes, most caffeine-degrading bacteria can utilize caffeine as their sole carbon and nitrogen source. *Pseudomonas* species, particularly *Pseudomonas Alcaligenes* CFR 1708 isolated from coffee grounds, have been extensively studied and exhibited high degradation efficiency, capable of completely degrading 1 g/L of caffeine within 4–6 h [12], while induced cells of *Pseudomonas* sp. demonstrated complete degradation of 1.2 g/L of caffeine within 6 h [91]. Fungal-mediated caffeine degradation capacity has also been investigated. For example, fermentation of Qingzhuan tea using *Penicillium simplicissimum* 4-17 for 21 days resulted in a 50% reduction in caffeine content while enhancing tea quality. Similarly, *Trichosporon asahii* was shown to degrade 60% of caffeine within three days [92]. In a comparative study of *R. oryzae*, *S. cerevisiae*, *Lactobacillus casei*, and *Leuconostoc mesenteroides*, *R. oryzae* demonstrated the highest caffeine reduction in coffee beans [15]. Currently, there is no standardized method to quantify the microbial degradation capacity of caffeine, which makes it challenging to directly compare different strains.

There are few studies that demonstrate large-scale degradation of caffeine through microbial processes or optimization techniques. Babu et al. [12] studied the decaffeination ability of immobilized *Pseudomonas alcaligenes* CFR 1708 cells. Through the optimization of bioreactor parameters such as pH, temperature, inoculum size, and caffeine concentration, complete degradation of 1 g/L caffeine was achieved within 4 to 6 h. Further studies investigated how initial caffeine concentration, dosing frequency, and substrate addition affect degradation kinetics. Using manual intermittent pulse dosing with an initial caffeine concentration of 3 g/L, the highest observed caffeine degradation rate was 0.82 g/L·h, along with a demethylase activity of 2.6 U/mg. The study remained the first to report the complete degradation of up to 237 g of caffeine within 75 h [93].

To enhance industrial applications of caffeine degradation, it is essential to select highly efficient strains and employ controlled bioreactor systems. These systems provide optimal growth conditions for microbial activity, ensuring process efficiency during fermentation.

#### 4.4.2. Alkaloid Tolerance of the Strain

During microbial caffeine degradation, high caffeine concentrations can inhibit the growth and reproduction of specific microorganisms. Therefore, isolating robust strains that exhibit both high caffeine tolerance and efficient degradation is crucial. To date, the most caffeine-tolerant microbes identified are predominantly Pseudomonas species. For example, *Pseudomonas putida* [11] and *Pseudomonas monteilii* KAJ 36 [94] can grow in media containing 20 g/L and 10 g/L caffeine, respectively, with degradation activities exceeding 80%. *Brevibacterium* sp., isolated from coffee pulp, showed lower tolerance to caffeine, with a maximum survivable concentration of 6 g/L in solid media and 4 g/L in liquid media [80]. These findings show that microbial caffeine tolerance varies greatly among species, indicating that the selection of degradation strains should be tailored based on the caffeine concentration present in the target substrate. However, high concentrations of caffeine can inhibit microbial metabolic activity. Most caffeine-degrading microbes thrive and perform best at caffeine concentrations below 5 g/L [95].

Identifying microbial strains that can tolerate high caffeine concentrations and efficiently degrade it within a short timeframe remains challenging. Most studies on microbial caffeine degradation involve culturing microorganisms in caffeine-supplemented media, followed by periodic measurements of caffeine concentration to assess metabolic activity. Researchers aim to enhance tolerance and degradation capacity in strains by gradually increasing the caffeine concentration in the medium, which can later be cultivated in bioreactors to improve metabolic performance. Some studies have also employed cell immobilization techniques to improve microbial caffeine tolerance. For instance, the immobilization of *Leifsonia* sp. SIU on agarose has been reported [96], and *Paenibacillus macerans* was immobilized in sodium alginate and NaCl [57]. Immobilized strains can be recovered and reused across multiple fermentation cycles, indicating a promising strategy for caffeine degradation.

Challenges remain in identifying strains that degrade caffeine, despite selective induction in culture media and enhancements via cell immobilization, particularly for low-caffeine coffee and tea production. These challenges arise from variations in substrate concentration and the complicated composition of real-world fermentation matrices, which can influence microbial activity and caffeine metabolism.

#### 4.4.3. Environmental Adaptability of Alkaloid-Degrading Microorganisms

The adaptability of microorganisms that degrade alkaloids is crucial for their use in food fermentation systems, such as coffee and tea processing. In real fermentation environments, microorganisms encounter various factors, including changing temperatures, pH levels, oxygen availability, osmotic pressure, and the presence of different inhibitory compounds. Microbial strains used for the degradation of caffeine and other alkaloids must retain their metabolic activity and viability under dynamic and often suboptimal conditions.

Fermentation conditions are closely linked to microbial performance and directly influence the quality of coffee fermentation. Studies on caffeine-degrading microorganisms such as *Aspergillus sydowii* NRRL250, *Aspergillus niger* [14], *Bacillus* sp. KS38 [43], and *Trichosporon asahii* [92] have shown that their optimal growth typically occurs at pH 6–7 and temperatures between 20–30 °C. Adding extra carbon sources like glucose can enhance caffeine degradation. In a comprehensive screening of 144 wild yeast strains, Pereira et al. assessed their tolerance to variations in pH, osmotic stress, heat, and the accumulation of metabolites, including ethanol (12–15%), lactic acid (2%), and acetic acid (2%). Stress-tolerant strains such as *Pichia fermentans*, *Pichia kluyveri*, *Pichia guilliermondii*, *Hanseniaspora opuntiae*, *Candida glabrata*, and *Saccharomyces* sp. were identified as promising candidates for challenging fermentation environments [97]. In addition to optimizing environmental conditions, microbes can adapt through internal metabolic regulation. For example, in yeast, the ADE gene is activated in response to acetic acid stress, resulting in the production of γ-aminobutyric acid (GABA), which acts as a stress protectant. To enhance microbial caffeine degradation under varying conditions, strategies such as bioreactor integration, cell induction, and immobilization can be employed to ensure stable and efficient fermentation processes.

#### 4.4.4. Safety and Quality of Low-Caffeine Coffee Products

Ensuring food safety and maintaining product quality are essential when using microorganisms in the production of low-caffeine coffee. To date, 121 microbial species capable of degrading caffeine have been identified. However, most of these studies have focused on bioremediation, specifically in treating water or soils contaminated with caffeine rather than on their application in food systems. The strains involved, mainly from the *Pseudomonas* genus, exhibit tolerance to low temperatures, high salinity, extreme pH levels, and alkaloids; yet their direct use in food is limited.

Only a small subset of microorganisms that degrade caffeine have been used in food-grade fermentation. For the production of low-caffeine tea, fungi such as *Aspergillus sydowii*, *Aspergillus niger*, *Candida famata* ACCC 2052, *Candida albicans* ACCC 2100, and *Penicillium simplicissimum* 4–17 have been used (Table 1). In low-caffeine coffee fermentation, six strains have been reported: *Aspergillus ochraceus*, *R. oryzae*, *S. cerevisiae*, *Leuconostoc mesenteroides*, *Lactobacillus casei*, and *Torulaspora delbrueckii* (Table 1). Several strains, including *A. niger*, *C. famata* ACCC 2052, *R. oryzae*, *S. cerevisiae*, *L. mesenteroides*, *L. casei*, and *T. delbrueckii*, are well-known food-grade fermentative microorganisms recognized for their flavor-enhancing properties and safety. Their safety profiles make them suitable candidates for low-caffeine coffee fermentation and provide a valuable reference for microbial strain selection.

Furthermore, *S. cerevisiae* has been demonstrated to produce citric acid during fermentation, which can inhibit the germination of *Aspergillus niger* spores, a known producer of ochratoxin. This suggests that organic acid production may play a role in suppressing undesirable microbes, thereby improving the microbiological safety of fermented coffee. These insights highlight the potential of mixed-culture fermentation strategies to lower caffeine content and improve product safety [98].

There are also reports of caffeine-degrading microorganisms growing on coffee by-products (Table 1), such as *Rhizopus* sp., *Pleurotus ostreatus*, and *Brevibacterium* sp. (MTCC 10313), indicating their potential caffeine metabolism capability. While *Pleurotus ostreatus* is an edible fungus with established safety for food applications, the safety profiles of the other strains remain uncertain. Comprehensive safety assessments are crucial if these microorganisms are to be used in producing low-caffeine coffee or tea. This includes evaluating pathogenicity, toxin production, and regulatory status before considering their application in food-grade fermentation.

Molecular biology techniques allow for the development of highly efficient caffeine-degrading strains. For example, the precise integration of N-demethylase genes (*ndmABCDE*) into *Bacillus amyloliquefaciens* HZ-12 has been demonstrated to enhance caffeine degradation efficiency by 21%, without generating endotoxins or off-odors [39]. This engineered strain (HZ-12: *ndmABCDE*) shows great promise for producing decaffeinated coffee and tea. However, as a genetically modified organism (GMO), it is still in the experimental phase due to regulatory and safety restrictions and has not yet been approved for commercial use. As noted earlier, the safety profiles and flavor impact of bacterial or fungal strains used in low-caffeine coffee production must be thoroughly assessed. A comprehensive approach is necessary to evaluate the effects of caffeine-reducing fermentation on product quality. This includes sensory analysis, nutritional composition changes, and volatile compound profile shifts. Among these factors, aroma plays a crucial role in consumer preference. Microbial fermentation has the potential to significantly modify and enhance the aromatic complexity of coffee.

Some microbes can negatively affect flavor. For instance, *Pseudomonas putida*, found in tea plantation soil, produces an unpleasant fishy odor, which is undesirable in fermented coffee [11]. Additionally, overfermentation can cause excessive acidification (pH < 4), negatively affecting the flavor and mouthfeel of the final product. Therefore, careful control of fermentation time and selection of strains is crucial for balancing caffeine reduction while maintaining or even enhancing coffee quality. Although currently the FDA/EFSA’s regulation of decaffeinated coffee mainly focuses on chemical solvent residues, the food-grade microbial safety standards (e.g., the QPS list established by the EFSA) are mandatory [30]. Only approved microbial species can be used for food fermentation. It is necessary to strictly test the metabolic products generated during the fermentation process and strictly control the fermentation process to ensure that the final product does not contain pathogenic pollutants or toxins.

The technology of producing decaf coffee through fermentation is still in the laboratory research stage. However, due to its characteristics of safety, low cost, simple equipment, and easy operation, small-scale farmers can adopt the circular bio-economy model to improve the feasibility of using this new technology. For example, using coffee pulp waste as a low-cost fermentation medium to form a green cycle. It can also simplify the fermentation equipment required, such as the application of plastic buckets, sealed bags, and other simple fermentation equipment instead of stainless steel fermentation tanks, reducing costs. 

## 5. Coffee Fermentation

### 5.1. Types of Coffee Fermentation

Controlled fermentation has become a key method for improving the quality of specialty coffee [17]. This process enhances the characteristics of coffee products available in the market. Researchers have developed a variety of fermentation devices designed for coffee cherries, taking into account different factors such as oxygen concentration, temperature, humidity levels, and the rate of microbial inoculation. The focus of coffee fermentation research has shifted from traditional dry, semi-dry, and wet methods to a more precise control of environmental conditions. Proper regulation of factors such as oxygen levels, humidity, and microbial communities during the fermentation process is essential for producing high-quality coffee. Fermentation mainly involves microbial breakdown of sugars into various metabolites, including ethanol, acetic acid, lactic acid, and butyric acid. Fermentation not only affects the acidity and flavor of coffee but also helps develop flavor precursors and alters the chemical composition of the coffee beans. Research on coffee fermentation primarily focuses on four key factors: the pre-treatment of coffee cherries, oxygen availability, humidity control, and the use of fermentation agents.

The quality characteristics of fermented coffee are closely linked to the methods used for post-harvest processing of coffee beans. Pre-fermentation treatment of coffee cherries typically involves either whole-fruit fermentation or depulping [99]. Naturally fermented coffees are typically noted for their higher acidity and sweetness, with malic acid being the predominant organic acid. In contrast, pulped coffees are mainly associated with profiles rich in citric acid. After depulping, the mucilage layer of the coffee fruit, which contains high levels of moisture and sugars, fosters a favorable environment for microbial activity. As a result, a pre-fermentation step is often implemented to inhibit the growth of undesirable microorganisms while enhancing the availability of beneficial fermentation by-products. Coffee cherries support a dynamic microbial community throughout the growth, maturation, and harvesting stages. Components such as cellulose, pectin, reducing sugars, lipids, proteins, organic acids, and alkaloids present in the fruit provide ample substrates for the bacteria, yeasts, and filamentous fungi involved in the fermentation process [100].

**Figure 3:**
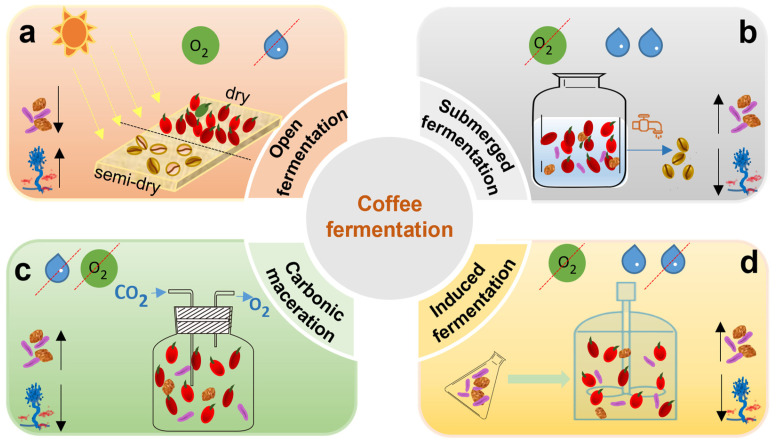
Types of fermentation of coffee beans. (**a**) Open fermentation. (**b**) Submerged fermentation (**c**) Carbonic maceration. (**d**) Induced fermentation.

Open-environment fermentation (Figure 3a) is a process that takes place under oxygen-rich conditions. This method spreads freshly harvested coffee cherries in a thin layer (5–10 cm) directly on the ground without removing the pulp. The cherries are continuously turned and dried until their moisture content decreases to approximately 12% [101]. During this drying period, natural fermentation occurs, driven by epiphytic microorganisms that break down the pectin and cellulose in the fruit’s skin. This allows microbial activity to penetrate the interior of the cherry and initiate a series of biochemical transformations. Microbial fermentation helps break down various compounds. Polysaccharides are converted into monosaccharides, proteins into amino acids, lipids into fatty acids, and chlorogenic acids into phenolic acids. In an aerobic environment, the abundant oxygen suppresses strictly anaerobic metabolic pathways. Instead, facultative microorganisms, like yeasts, play a critical role as they can use both respiratory and fermentative processes. However, this open environment, which is characterized by high temperatures, humidity, and sugar content, may also encourage the growth of fungi such as *Aspergillus*, *Fusarium*, and *Penicillium* sp. These fungi can produce butyric acid, propionic acid, and mycotoxins, which may result in off-flavors and reduced coffee quality [18]. To reduce these risks, microbial inoculation for controlled pre-fermentation before drying has been suggested, along with the strict regulation of temperature and humidity. Notably, pre-fermentation using inoculated *S. cerevisiae* has been demonstrated to significantly improve the sensory qualities of the final coffee product [102], indicating that high-quality specialty coffee production is feasible even under open-environment conditions.

Submerged fermentation [103], as illustrated in Figure 3b, is frequently used in areas with high humidity, such as Colombia and Hawaii. In this method, freshly harvested coffee cherries are depulped and placed in water tanks, where naturally occurring microbial communities conduct the fermentation process. This technique effectively removes the mucilage layer from the coffee beans. This wet processing typically boosts fruity aromas while minimizing burnt or bitter flavors. The fermentation period typically lasts between 12 and 72 h. Extended fermentation can result in a smoother body and a more complex acidity, which is particularly appealing to consumers who favor bright, clean cup profiles. Nonetheless, the success of wet fermentation relies on various factors, such as the ripeness of the cherries, temperature, humidity, and the composition of the microorganisms involved. High temperatures can result in undesirable off-flavors in coffee, such as vinegar-like or metallic tastes. One advantage of wet fermentation is that it facilitates easy stirring of the beans, which promotes uniformity and enhances the removal of mucilage. This process creates a microaerobic environment that enhances microbial diversity. It encourages the growth of various microorganisms, including LAB, acetic acid bacteria, *Bacillus* species, yeasts, and filamentous fungi. These microorganisms play a crucial role in producing volatile compounds such as alcohols, esters, organic acids, ketones, and aldehydes, which together improve the sensory profile of the final coffee product [104]. Prolonged wet fermentation, lasting more than 48 h, can lead to a reduction in acidity and low-molecular-weight sugar content, negatively impacting coffee quality. By inoculating microbial strains in bioreactors, fermentation time can be significantly shortened while maintaining product consistency. External inoculation with *S. cerevisiae* and *L. plantarum* has been shown to encourage the formation of desirable volatile compounds [17]. Various microorganisms have been identified in wet fermentation. These include filamentous fungi belonging to *Penicillium*, *Aspergillus*, and *Fusarium*, as well as yeasts like *S. cerevisiae* and *Pichia fermentans*. Yeast activity not only prevents the growth of aerobic filamentous fungi and the formation of off-flavor compounds such as butyric and acetic acids but also boosts antioxidant capacity. Furthermore, yeast activity increases the levels of alcohols, esters, aldehydes, glycerol, and organic acids in fermented beans [105]. *S. cerevisiae* and *Pichia fermentans* are commonly used yeast inoculants in coffee fermentation, while *L. plantarum* represents a key bacterial species in this process. The activity of pectinolytic enzymes produced by both yeasts and bacteria is a critical factor, as it directly affects the efficiency of mucilage removal. In summary, using starter cultures during wet fermentation can improve process efficiency and ensure consistent product quality. However, when implementing deep wet fermentation with these inoculants, it is essential to manage the process carefully to prevent contamination by aerobic microorganisms and other unwanted microbes.

Carbonic maceration (CM) and semi-carbonic maceration fermentation (Figure 3c) are anaerobic fermentation techniques adapted from winemaking [106]. In oxygen-free conditions, microbial metabolism in the cytoplasm converts sugars into ethanol, carbon dioxide, glycerol, pyruvate, succinic acid, and various volatile alcohols. The anaerobic environment produced by carbon maceration of coffee fruits inhibits the aerobic respiration process. The microbial community during the coffee fermentation process changes over time. After 120 h of fermentation at 38 °C, the fungal diversity was the highest [107], and the yeast *Pichia cephalo-cereana* is dominant during the fermentation process [108]. Although research on CM in coffee is still limited, it points that maintaining the fermentation temperature at 36 °C could reduce the overall fermentation duration by more than 4 days [109]. During coffee fermentation in sterile plastic bags filled with carbon dioxide, an increase in microbial diversity was observed over time. This increase correlated positively with improvements in aroma and aftertaste scores. Additionally, the metabolites trigonelline and formic acid were found to increase, suggesting a potential link between carbon dioxide fermentation and enhanced coffee quality. This finding warrants further investigation [106]. While the anaerobic environment inhibits the growth of pathogenic aerobic microorganisms, the wider use of CM in coffee is limited by the requirement for reactors that manage pressure and CO_2_ levels, leading to higher operational costs. Consequently, CM remains relatively underexplored in coffee fermentation research.

Self-induced anaerobic fermentation (SIAF) (Figure 3d) [110] is a type of solid-state fermentation carried out in an oxygen-restricted environment, where controlling temperature is essential for managing the fermentation duration. This method involves introducing specific microbial strains to enhance fermentation performance, reduce contamination by undesirable microbes, and increase the production of beneficial compounds such as volatile acids, lactones, phenols, pyrans, pyrazines, pyridines, and thiophenes. These compounds significantly improve the sensory quality of coffee beverages. When *S. cerevisiae* was inoculated under SIAF conditions, the resulting coffee displayed strong notes of chocolate, caramel, and floral aromas, with sensory scores exceeding those of naturally fermented coffees that were later inoculated with yeast [111]. In SIAF, the predominant microbial genera are *Enterobacter*, *Lactobacillus*, *Pantoea*, and *Cladosporium*. These genera enhance fruitiness and sweetness, increasing overall sensory scores by up to 3.8 points [112]. Cassimiro et al. combined sequential inoculation and SIAF with wet fermentation, using co-inoculation of *L. plantarum* and *S. cerevisiae* [17]. They observed high microbial carbohydrate consumption and the production of volatile compounds like 2,3-butanediol, which contributed to improved coffee quality scores. Additionally, SIAF has been shown to increase lactic acid concentration, alter volatile compound profiles, and create coffees with more pronounced fruity characteristics [113]. Integrating controlled bioreactors and specific starter cultures into the SIAF process presents significant potential for enhancing the standardized production of high-quality specialty coffee.

### 5.2. Microbial Interaction Mechanisms in Coffee Fermentation

Specialty coffee is a high-quality beverage recognized for its distinctive flavors and sensory qualities. The Specialty Coffee Association (SCA) defines specialty coffee as coffee that scores at least 80 out of 100 in various sensory parameters. These parameters include aroma, flavor, aftertaste, acidity, body, uniformity, balance, sweetness, cleanliness, and overall impression. Exogenous microbial inoculation offers several benefits during coffee fermentation. It can shorten fermentation time, inhibit harmful microorganisms, and enhance the interaction between microbial activity and the natural metabolites in the coffee beans. These interactions may result in unique precursor compounds that permeate the beans, enhancing the overall quality. Therefore, microbial fermentation can play a crucial role in controlling coffee quality. Inoculating coffee with selected microbes can enhance its characteristics by speeding up the fermentation process, inhibiting undesirable microbes, and encouraging the formation of flavor precursors [114]. The key agents of fermentation are yeasts and LAB, which interact in either a supportive or competitive way to regulate metabolic pathways and influence the flavor profiles of coffee.

Yeasts play a key role in shaping the microbial community during coffee fermentation by breaking down carbohydrates, organic acids, and volatile compounds found in the pulp and mucilage, thereby changing the flavor profile of the green beans through metabolic exchanges. The aromatic esters produced by yeasts add fruity and floral notes to coffee. Furthermore, yeasts inhibit the growth of filamentous fungi, which improves the safety of the fermentation process. Common yeast genera involved in coffee processing include *Pichia*, *Candida*, *Saccharomyces*, and *Torulaspora*. Notably, *Pichia kudriavzevii* is tolerant of high temperatures and acidity, which supports the development of desirable flavor characteristics [115]. *T. delbrueckii* exhibits pectinolytic activity and contributes caramel and floral aromas [100]. *Pichia fermentans* and *Saccharomyces* sp. are dominant yeast strains suitable for fermentation starter cultures [116]. Yeast has been used to ferment coffee from seven regions in Brazil. It was discovered that yeast could impart unique flavors to coffee, such as coriander and jasmine. The sensory scores of flower, mint, red fruit jelly, candy corn, and crystallized orange increased significantly to 86–92.5 [110]. In the practical application of wet processing on a farm, *Pichia fermentans* YC5.2 proved to be conducive to the production of high-quality coffee with unique characteristics, such as a strong perception of vanilla flavor and a floral aroma [117].

LAB are vital microorganisms in the wet fermentation process. They significantly contribute to flavor development by producing lactic acid, regulating acidity, and breaking down mucilage. Through their metabolic pathways, including the breakdown of citrate and amino acids, they produce malic and tartaric acids, thereby enhancing fruity, floral, and caramel–honey aromas [118]. LAB also suppress contaminant microbes by lowering pH and interact with other species, such as yeasts, to stabilize the fermentation environment [119].

Co-inoculation of yeasts and LAB is a common strategy used in coffee fermentation. The interactions between these microorganisms, whether synergistic or competitive, significantly influence the distribution of metabolites. Organic acids produced by LAB lower the pH, which promotes yeast growth. Conversely, amino acids released by yeasts during their exponential growth phase support the metabolism of LAB [120]. *P. fermentans*, when co-inoculated with *P. acidilactici*, accelerated sugar consumption and increased the production of ethanol and ethyl acetate [121]. In another study, co-cultivating *B. licheniformis* with *M. guilliermondii* promoted the proliferation of LAB [122]. Similarly, yeasts were also able to enhance the survival of LAB during storage [20], indicating long-term advantages of mixed-species fermentation for microbial community stability.

### 5.3. Mixed-Culture Coffee Fermentation

Mixed-culture fermentation significantly enhances coffee quality by utilizing the synergistic interactions among various microbial strains. Different combinations of these microbes lead to distinct flavor profiles (Table 2). The most common method involves co-inoculating yeasts and LAB, as their complementary metabolic pathways enhance flavor complexity. Yeasts convert sugars into ethanol, while LAB further metabolize sugars and ethanol into lactic acid, which also helps ensure microbial safety. For instance, co-inoculation with *L. plantarum* CCMA1065 and *S. cerevisiae* CCMA0543 adds citrus and floral notes to coffee, while also increasing sweetness, acidity, and body [17]. Delayed yeast inoculation (48–96 h) can enhance fruity aromas, but it requires careful management of fermentation duration to prevent excessive metabolism [123]. Fermentation using *L. rhamnosus* GG and *Saccharomyces boulardii* CNCM-I745 leads to the accumulation of beneficial metabolites, including 2-isopropylmalic acid and aromatic amino acid derivatives. Research has shown that anaerobic co-inoculation of *S. cerevisiae* with *Bacillus amyloliquefaciens* can achieve SCA scores exceeding 84 points, which meets specialty coffee standards. Other effective combinations include *Pichia fermentans* YC5.2 with *Pediococcus acidilactici*, as well as *Leuconostoc mesenteroides* with *Torulaspora delbrueckii*. These combinations produce a diverse array of flavors, such as honey, caramel, chocolate, and floral notes (Table 2). These findings demonstrate that mixed-culture fermentation, facilitated by microbial interactions and optimized metabolic pathways, is an effective strategy for precisely modulating the sensory attributes of coffee.

Mixed-yeast inoculation is a common strategy used in coffee fermentation (Table 2). Yeasts consume sugars found in the coffee pulp, such as fructose, glucose, and sucrose, as their carbon sources. This process decreases the sweetness of the coffee while also influencing its acidity. Yeasts achieve this by utilizing the tricarboxylic acid (TCA) cycle to break down citric, malic, and succinic acids [129]. Research has demonstrated that co-inoculation with *Wickerhamomyces anomalus* YN5 and *Kazachstania humilis* YN9 increases the antioxidant activity of coffee pulp. This process also enhances the release of flavor compounds through ethanol production, resulting in floral, fruity, and sweet liqueur-like aromas in the coffee [130]. The fermentation of Robusta coffee (*C. canephora*) is significantly enhanced by the use of *S. cerevisiae*, which improves both fermentation efficiency and sensory evaluation scores [131]. Both *Saccharomyces* and *Pichia* species aid in releasing phenolic compounds from cellulose, allowing metabolites to penetrate the bean and enhancing overall quality [132]. A triple inoculation of *S. cerevisiae* CCMA 0543, *Candida parapsilosis* CCMA 0544, and *Torulaspora delbrueckii* CCMA 0684 yielded a sensory score of 86.9. This combination produced a complex flavor profile with notes of caramel and mint, rose and honey, and woody floral hints. While yeast fermentation enhances phenolic content and antioxidant activity, the depletion of sugars and accumulation of organic acids can lead to increased acidity and reduced sweetness, which may affect consumer acceptance. Yeast co-inoculation was carried out on coffee beans from the low-altitude Caparao region (Guacui-ES Brazil). It was found that the co-inoculation of *C. parapsilosis* CCMA 0544 and *T. delbrueckii* CCMA 0684 could significantly improve the sensory score of coffee, presenting a distinct cocoa/chocolate, caramel, peppermint, rose-honey-like floral -woody [125]. Research on the co-inoculation of pure LAB is limited. However, studies have shown that *Leuconostoc mesenteroides* and *L. plantarum* can produce caramel, fruity, and spicy flavors during SIAF. In general, different mixed-culture fermentation strategies improve the sensory quality and flavor complexity of coffee by leveraging microbial synergy. This provides a strong foundation for further optimizing the coffee fermentation process.

### 5.4. Optimization of Mixed-Culture Coffee Fermentation

**Figure 4:**
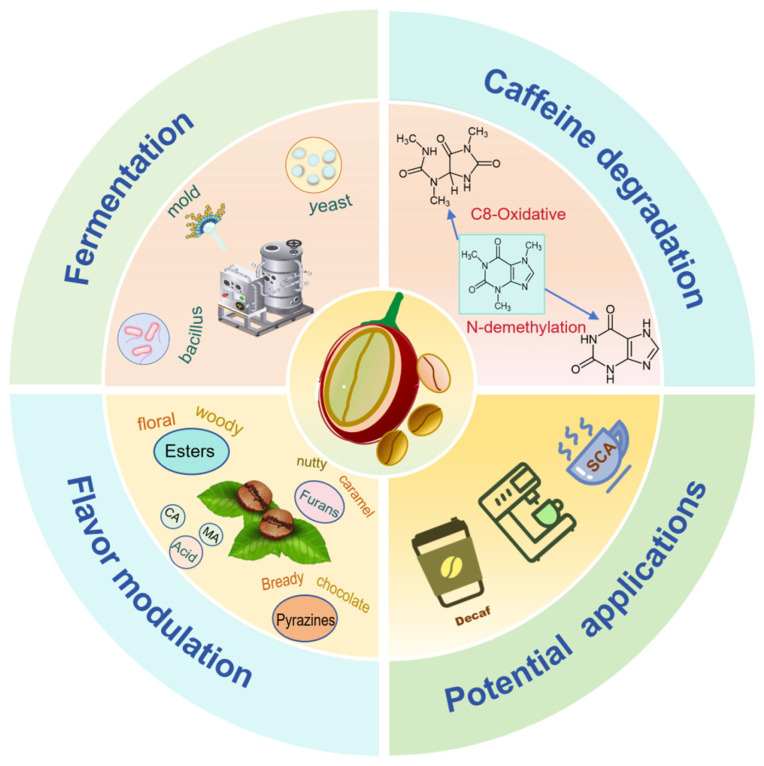
Effect of caffeine degradation and mixed fermentation on coffee quality.

Sequential inoculation (Figure S1) effectively manages microbes and enhances synergy during mixed-culture coffee fermentation. Unlike simultaneous inoculation, this staggered approach minimizes nutrient competition among microbial strains and enables the primary inoculant’s metabolic activity to establish favorable conditions for the subsequent strain. Previous studies have summarized that in fermentation systems with LAB and *Pseudomonas*, simultaneous inoculation could significantly inhibit the growth of *Pseudomonas taetrolens* due to the antimicrobial compounds produced by LAB. However, this issue was effectively mitigated by delaying the inoculation of LAB in the sequential strategy [133]. The interval between inoculations is crucial because a proper delay allows the first strain (such as LAB) to lower the pH and modify the environment, facilitating the growth and metabolic activity of the second strain [134]. Sequential inoculation plays a crucial role in preventing over-fermentation, which otherwise can result in the growth of spoilage bacteria and a loss of important flavor compounds. By managing the duration and temperature of fermentation, this approach can improve the development of esters, ketones, and other desirable volatile compounds. For instance, using *Lachancea thermotolerans* followed by *O. oeni* Lalvin promotes stable growth of *O. oeni*, prevents premature yeast death, reduces acidity and bitterness, and preserves a wider range of complex volatile aroma compounds [135]. Hence, combining precise environmental regulation with bioreactors (Figure 4) and sequential inoculation enhances the quality and consistency of coffee fermentation. These insights provide a solid theoretical foundation and technical support for optimizing coffee fermentation processes.

## 6. Conclusions and Future Perspectives

This article reviews the diversity and mechanisms of alkalinity reduction among caffeine-degrading microorganisms, with a particular focus on those utilized in the processing of coffee beans and tea. It provides a comparative analysis of the advantages and limitations of various decaffeination methods used in coffee production. Furthermore, this study elucidates the regulatory influence of microbial metabolism on coffee bean flavor through an analysis of the synergistic effects observed in mixed-culture fermentation systems. Current research primarily focuses on caffeine removal from coffee by-products such as pulp and husk, while there is limited investigation directed at reducing caffeine content in the coffee bean itself. To date, six microbial strains capable of degrading caffeine in coffee beans have been identified. Among these, five are classified as food-safe, while the exception is *Aspergillus ochraceus*. However, several significant challenges still impede practical implementation. One of the most notable issues is the alkaloid tolerance of caffeine-degrading microbes, which limits their efficiency in degrading caffeine. Caffeine degradation mainly occurs through two metabolic pathways: N-demethylation and C-8 oxidation. The N-demethylation pathway has been studied the most extensively, as it not only effectively reduces caffeine levels but also produces secondary metabolites that may have functional and economic value. These properties create new opportunities for valorizing coffee-processing by-products and support the development of environmentally friendly “waste-to-value” production models. However, potential safety concerns, such as the formation of mycotoxins during microbial fermentation, necessitate systematic evaluation. Future research should emphasize the following: (1) improving microbial tolerance to caffeine through adaptive evolution to enhance degradation efficiency; (2) developing mixed-culture fermentation systems that combine caffeine-degrading strains with flavor-enhancing microorganisms (e.g., yeasts, LAB) to simultaneously achieve decaffeination and sensory quality improvement; (3) deciphering the molecular mechanisms of caffeine degradation using integrated multi-omics approaches (genomics, transcriptomics, metabolomics, proteomics); (4) establishing comprehensive food safety evaluation frameworks to ensure the safety of fermented products for human consumption; and (5) exploring intelligent fermentation control technologies for the precise regulation of the decaffeination process. Consumer acceptance of decaf coffee shows a significant upward trend. In the future, joint brand marketing can be utilized to enhance the flavor experience and expand the decaf coffee market. With ongoing research and advancements in technology, microbial fermentation is anticipated to address the limitations of traditional decaffeination methods. This innovative approach could allow for the creation of unique low-caffeine coffee products that maintain their characteristic flavor while minimizing health risks. The development of this technology not only promotes a sustainable transformation within the coffee industry but also offers a valuable strategy for enhancing other caffeine-containing foods.

## Figures and Tables

**Table 2:** Microbial combinations for the mixed fermentation of coffee beans.

Types	Mixed Fermentation	Fermentation/Inoculation Mode	Flavor Characteristics	References
Yeast + Bacteria	*L. plantarum + S. cerevisiae*	Sequential inoculation/wet	Greater acidity, sweetness, body	[123]
	*Lactobacillus rhamnosus* GG+ *Saccharomyces boulardii* CNCM-I745	Simultaneous inoculation/wet	_	[124]
	*S. cerevisiae + Bacillus amyloliquefaciens*	Simultaneous inoculation/SIAF	Nutty, cocoa, and sweet	[115]
	*Pichia fermentans* YC5.2 *+ Pediococcus acidilactici* LPBC161	Combined fermentation/wet	Honey, floral, sweet and grass fragrance	[121]
	*Leuconostoc mesenteroides + Torulaspora delbrueckii*	SIAF/wet	Chocolate, wheat, green, woody, floral, caramel,	[1]
	*Leuconostoc mesenteroides + S. cerevisiae*	SIAF/wet	Chocolate, caramel, fruity, spices	
	*L. plantarum + Torulaspora delbrueckii*	SIAF/wet	Chocolate, dark chocolate, nutty, fruity, spices	
	*L. plantarum + S. cerevisiae*	SIAF/wet	Dark chocolate, caramel, nutty, spices	
	*L. plantarum* CCMA1065 *+ S. cerevisiae* CCMA0543	Co-inoculation/wet	Citrus, floral	[17]
	*Bacillus licheniformis + Meyerozyma guilliermondii*	Co-inoculation/wet	Almond, chocolate	[122]
Yeast + Yeast	*S. cerevisiae* CCMA 0543 *+ Candida parapsilosis* CCMA 0544	Co-inoculation/dry	Fruity, nutty, roasted, sweet, almond, biter, cocoa, coffee, roasted nutty, caramel and peppermint, rose–honey-like floral–woody	[125]
	*S. cerevisiae* CCMA 0543 *+ Torulaspora delbrueckii* CCMA 0684	Co-inoculation/dry	Fruity, caramel and peppermint, rose–honey-like floral–woody	
	*Candida parapsilosis* CCMA 0544 *+ Torulaspora delbrueckii* CCMA 0684	Co-inoculation/dry	Caramel and peppermint, rose–honey-like, floral–woody	
	*Torulaspora delbrueckii* *+ Candida parapsilosis*	SIAF/wet	Citrus, caramel, honey, chocolate, chestnut	[126]
	*S. cerevisiae* CCMA 0543 *+ Candida parapsilosis* CCMA 0544 *+ Torulaspora delbrueckii* CCMA 0684	Co-inoculation/dry	Caramel and peppermint, rose–honey-like, floral–woody, citric acidity, fruity, banana and pear	[46]
	*Hansinaspora uvarum +Pichia kudriavzevii*	Combined fermentation/wet	Earthy, apple cider, walnut notes, smooth mouthfeel	[127]
	*S. cerevisiae* CCMA 0543 *+ Candida parapsilosis* CCMA 0544 *+ Torulaspora delbrueckii* CCMA 0684	SIAF/wet	Caramel, peppermint, citric acidity	[128]
Bacteria + Bacteria	*Leuconostoc mesenteroides + L. plantarum*	SIAF/wet	Caramel, fruity, spices	[1]

## Data Availability

The original contributions presented in this study are included in the article/Supplementary Materials. Further inquiries can be directed to the corresponding author.

## Supplementary Materials

The following supporting information can be downloaded at https://www.mdpi.com/article/10.3390/foods14152606/s1: Supplementary Table S1: Enzymes related to caffeine degradation; Supplementary Figure S1:Fermentation inoculation method;
