# Supplementary material for: Application of Microbial Fermentation in Caffeine Degradation and Flavor Modulation of Coffee Beans
Source: Foods. 2025 Jul 24;14(15):2606. doi: 10.3390/foods14152606 (PMC12346622; doi:10.3390/foods14152606)

(Supplementary Material)

Application of Microbial Fermentation in Caffeine Degradation and Flavor Modulation of Coffee Beans

Lu-Xia Ran [1,2,3,†], Xiang-Ying Wei [1,2,3,†], Er-Fang Ren [1], Jian-Feng Qin [1], Usman Rasheed [1,2,3] and Gan-Lin Chen [1,2,3,*]

1 Guangxi Subtropical Crops Research Institute, Guangxi Academy of Agricultural Sciences, Nanning 530001, China; 1211016008@gnnu.edu.cn (L.-X.R.); 15102932953@163.com (X.-Y.W.); aabbc159@163.com (E.-F.R.); qjfmail@126.com (J.-F.Q.); rasheus@outlook.com (U.R.)

2 Key Laboratory of Quality and Safety Control for Subtropical Fruit and Vegetable, Ministry of Agriculture and Rural Affairs, Nanning 530001, China

3 Guangxi Key Laboratory of Quality and Safety Control for Subtropical Fruits, Nanning 530001, China

* Correspondence: ganlin-chen@163.com or ganlin-chen@gxaas.net; Tel.: +86-771-2539099

† These authors contributed equally to this work.

Supplementary Table S1: Enzymes related to caffeine degradation

Caffeine degradation pathway	Degrading enzyme	Function	Source	References
N-demethylation	NdmA	N1-demethylase specific for N1-methyl group of caffeine	*Pseudomonas putida* CBB5	[1]
	NdmB	N3-demethylase specific for N3-methyl group of theobromine		
	NdmC	N7-demethylase specific for N7-methyl		
	NdmD	Eductase		
	NdmE	Provide structural support		
	NdmCDE	protein complex containing N7-demethylase specific for N7-demethylation of 7-methylxanthine		
C-8 oxidation	TmuM	Trimethyluric acid monooxygenase	*Pseudomonas* sp. CBB1	[2]
	TmuH	Putative TM-HIU hydrolase		
	TmuD	Putative TM-OHCU decarboxylase		
	cdh	Trimeric caffeine dehydrogenase		

[1] Summers, R.M.; Louie, T.M.; Yu, C.L.; Subramanian, M. Characterization of a Broad-Specificity Non-Haem Iron N-Demethyl ase from *Pseudomonas Putida* CBB5 Capable of Utilizing Several Purine Alkaloids as Sole Carbon and Nitrogen Source. *Microbi ology* **2011**, 157, 583–592, doi:10.1099/mic.0.043612-0.
[2] Mohanty, S.K.; Yu, C.-L.; Das, S.; Louie, T.M.; Gakhar, L.; Subramanian, M. Delineation of the Caffeine C-8 Oxidation Pathway in *Pseudomonas* Sp. Strain CBB1 via Characterization of a New Trimethyluric Acid Monooxygenase and Genes Involved in Trimethyluric Acid Metabolism. *J Bacteriol* **2012**, 194, 3872–3882, doi:10.1128/JB.00597-12.

Supplementary Figure S1:Fermentation inoculation method

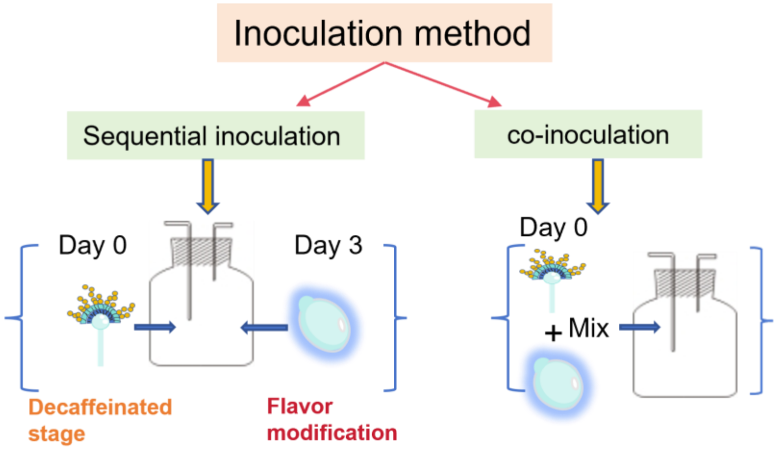

Supplement: Supplementary file 1 [file foods-14-02606-s001.zip › foods-3723888-supplementary.pdf]
